# Imidacloprid transport and sorption nonequilibrium in single and multilayered columns of Immokalee fine sand

**DOI:** 10.1371/journal.pone.0183767

**Published:** 2017-08-24

**Authors:** Jorge A. Leiva, Peter Nkedi-Kizza, Kelly T. Morgan, Davie M. Kadyampakeni

**Affiliations:** 1 Soil and Water Sciences Department, University of Florida, Institute of Food and Agricultural Sciences (UF-IFAS), Gainesville, United States of America; 2 UF-IFAS Southwest Florida Research and Education Center, Immokalee, United States of America; 3 UF-IFAS Citrus Research and Education Center, Lake Alfred, United States of America; Universidade Estadual Paulista Julio de Mesquita Filho, BRAZIL

## Abstract

Imidacloprid (IMD) is a neonicotinoid pesticide soil-drenched to many crops to control piercing-sucking insects such as the Asian citrus psyllid (ACP). Neonicotinoids are persistent in the environment and transport analyses are helpful estimate leaching potential from soils that could result in groundwater pollution. The objective of this study was to analyze IMD breakthrough under saturated water flow in soil columns packed with three horizons (A, E, Bh) of Immokalee Fine Sand (IFS). Also, we used the dimensionless form of the convective-dispersive model (CD-Model) to compare the optimized transport parameters from each column experiment (retardation factor, *R*; fraction of instantaneous-to-total retardation, *β*; and mass transfer coefficient, *ω*) with the parameters obtained from sorption batch equilibria and sorption kinetics. The tracer (Cl^-^) breakthrough curves (BTCs) were symmetrical and properly described by the CD-Model. IMD BTCs from A, Bh, and multilayered [A+E+Bh] soil columns showed steep fronts and tailing that were well described by the one-site nonequilibrium (OSNE) model, which was an evidence of non-ideal transport due to IMD mass transfer into the soil organic matter. In general, IMD was weakly-sorbed in the A and Bh horizons (*R* values of 3.72 ± 0.04 and 3.08 ± 0.07, respectively), and almost no retardation was observed in the E horizon (R = 1.20 ± 0.02) due to its low organic matter content (0.3%). Using the HYDRUS-1D package, optimized parameters (*R*, *β*, *ω*) from the individual columns successfully simulated IMD transport in a multilayered column mimicking an IFS soil profile. These column studies and corresponding simulations agreed with previous findings from batch sorption equilibria and kinetics experiments, where IMD showed one-site kinetic mass transfer between soil surfaces and soil solution. Ideally, sandy soils should be maintained unsaturated by crop irrigation systems and rainfall monitoring during and after soil-drench application. The unsaturated soil will increase IMD retardation factors and residence time for plant uptake, lowering leaching potential from soil layers with low sorption capacity, such as the E horizon.

## Introduction

**Fig 1:**
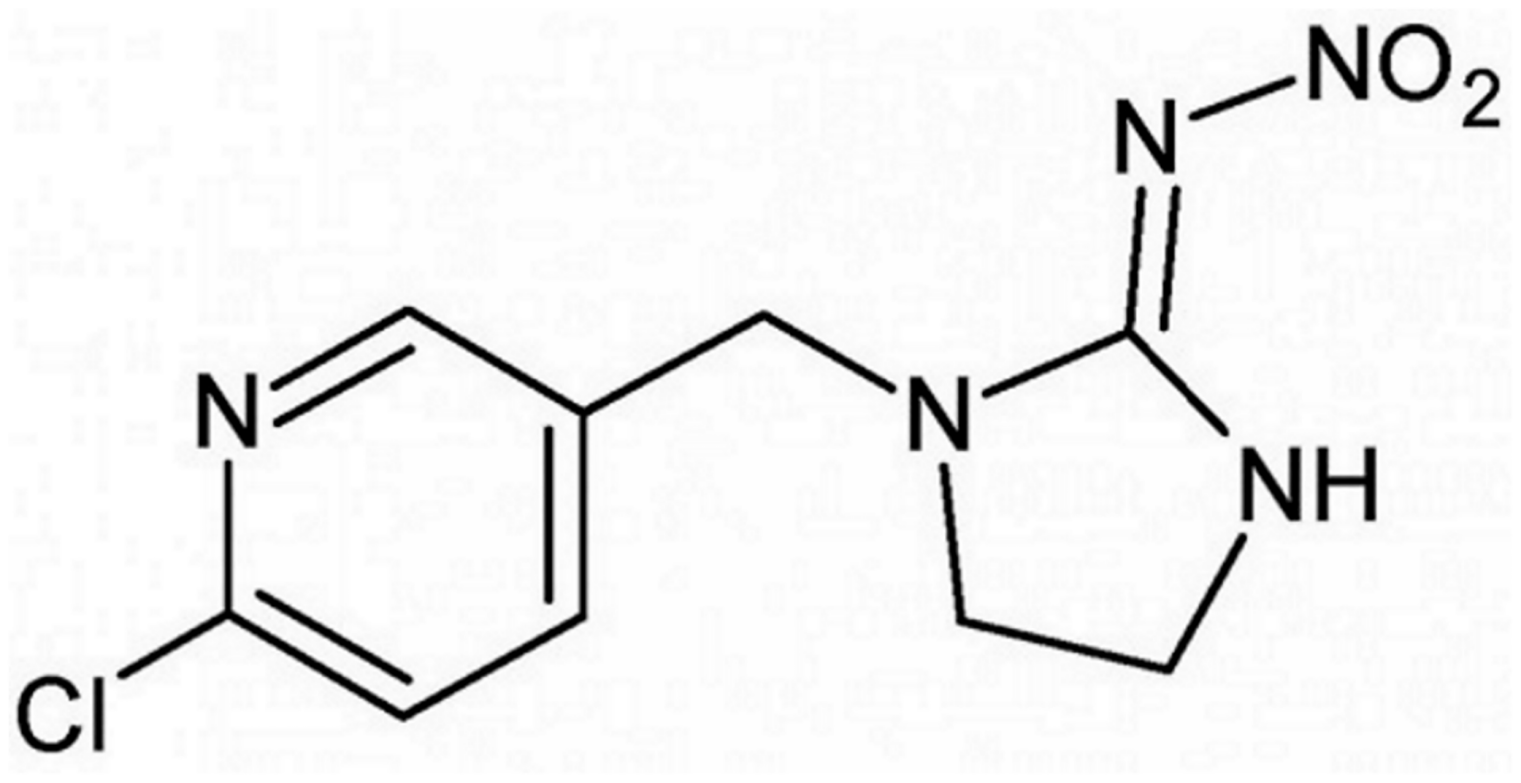
Imidacloprid molecular structure (6).

Imidacloprid (IMD, Fig 1) is a neonicotinoid insecticide (a synthetic derivative of nicotine) whose organic molecule is fairly soluble in water [1, 2], and it is considered one of the most widely used pesticides in the world [3–5]. The systemic properties of IMD allow absorption by plant roots and translocation to tender shoots and leaves where piercing-sucking insects, such as aphids and psyllids, feed upon [6]. IMD mode of action is by direct ingestion, and it interferes with stimulus between nervous cells, which paralyzes and kills the insect [7].

In Florida, IMD is applied to control the Asian Citrus Psyllid (ACP) *Diaphorina citri* (Kuwayama) [8, 9]. The ACP is the primary vector of the devastating citrus greening disease in many parts of the world [10]. ACP management in citrus production areas recommend that IMD should be applied as a seed treatment in nurseries, or as a soil-drench to young trees before blooming starts. The goal of this practice is to avoid killing important pollinators and other beneficial insects, such as bees and ladybeetles [11–13]. Therefore, direct applications of IMD to the soil surface (and citrus root zone) to control the ACP generate questions about uptake efficiency by the crop, persistency within the root-zone, and potential leaching into groundwater in landscapes such as the Florida Central Ridge and Florida Flatwoods, where most citrus commodities are grown. In fact, IMD was found at μg L^-1^ levels in 13% of the groundwater monitoring wells, where sandy soils with low organic matter dominate the landscape in Florida [14, 15]. It is important to understand IMD soil sorption and transport patterns in these soils to promote management practices that reduce potential for IMD leaching below the citrus root-zone.

Unfortunately, neonicotinoid class pesticides such as IMD, Acetamiprid, Thiamethoxam (TMX), Nitenpyram, Clothianidin, Dinotefuran (DTN), and Thiacloprid, have limited transport data from soil columns in the literature [3]. For instance, in Louisiana-soil columns IMD showed sorption nonequilibrium explained by a multi-reaction transport model that accounted for both reversible and irreversible sorption [16]. IMD showed moderate-to-high mobility largely explained by the soil organic matter content, clay mineralogy, and dissolved organic carbon content (DOC) [16]. In another study conducted in calcareous soils from Spain, IMD mobility was assessed in terms of the soil DOC content [17]. The experiment showed that DOC reduced IMD retardation and increased its leaching potential [17]. Also, a recent soil column study comparing pulse solutions with individual-solute and mixed-solutes showed that leaching potential for selected neonicotinoids followed IMD < TMX < DTN, with their mobility strongly correlated with the pesticide’s solubility in water [18]. Furthermore, our data on IMD soil batch kinetics and equilibria, as well as degradation rates in sandy soils of Florida showed that IMD had a moderate-to-weak sorption and persistence in these soils [19]. Those batch sorption studies showed that IMD kinetics were described by the one-site nonequilibrium mass transfer model [19, 20] which postulates that all sorption sites are kinetic or type-2 sites [21, 22]. One-site mass transfer kinetics have been reported for other organic solutes in soils, such as naphthalene [23]; atrazine and diuron [23, 24]; N-heterocyclic compounds, such as toluene and quinolone [25]; and, silver nanoparticles coated with polyvinyl-pyrrolidone [26].

Environmental studies of pesticide sorption and leaching in soil environments are costly endeavors [27, 28]. Miscible displacement of solutes through soil columns or breakthrough curves (BTCs) are used to analyze dynamic transport processes in porous media [29, 30]. BTC analysis is an applied-technique that is still widely used to estimate contaminant leaching potential and potential negative effects on groundwater quality [31, 32]. Also, pesticide early breakthrough and tailing are evidence of sorption kinetics by physical or chemical nonequilibrium processes [29, 33]. Retardation factors and mass transfer coefficients generated from BTCs are useful in pesticide transport modeling in the vadose zone [34–36].

The main objective of this study was to analyze the transport of IMD in soil columns packed with representative soil horizons from a Florida flatwoods soil (classified as Spodosols or Podzols) with contrasting physico-chemical properties. The study compared the optimized transport parameters from the individual soil horizons to the parameters obtain from sorption equilibria and sorption kinetics conditions. The transport parameters obtained from individual layers or columns were used to validate the OSNE solute transport model for IMD in a multilayered soil column that mimicked a soil profile. Saturated flow conditions were emphasized to assess IMD potential to leach below the citrus root-zone under worst case scenario conditions, which could occur during storm events in Florida. Analyzing IMD displacement and sorption phenomena in sandy soils of Florida will generate valuable data to improve ACP management practices, increase the efficacy of IMD soil-drench field applications in citrus groves, and avoid pesticide leaching and pollution of groundwater resources.

## Materials and methods

### Soil sampling and characterization

This research study was conducted under laboratory conditions at the University of Florida (UF), Gainesville. The laboratories are supervised by the UF’s Environmental Health and Safety Office. This study did not involve endangered or protected species.

The study was conducted with soil samples from Immokalee find sand (IFS), a Spodosol classified as sandy, siliceous, hyperthermic, Arenic Haplaquods [37]. The samples were collected from an area with secondary-growth flatwoods, at the University of Florida, Institute of Food and Agricultural Sciences (UF-IFAS), Southwest Florida Research and Education Center, Immokalee (latitude, 26° 27.75′ N; longitude, 81° 26.83′ W). The samples were collected from a soil pit and separated into three diagnostic horizons (Table 1) based upon Soil Taxonomy procedures [38]: surficial horizon A (ochric), and subsurface horizons E (albic), and Bh (spodic). Undisturbed soil core samples were also collected from each horizon to measure soil bulk density. The samples were transported in coolers to the UF-IFAS Environmental Soil Physics Laboratory in Gainesville, Florida, where they were air-dried for one week, sieved (<2 mm), and mixed thoroughly in buckets before column packing. Subsamples from the mixed air-dried soils were taken before column packing to determine moisture content and to obtain bulk density (*ρ*_*b*_) values such as those observed in the field. Soil particle density (*ρ*_*S*_) was determined by the pycnometer method [39] and organic matter content was measured using the loss-on-ignition method [40]. Methods to determine soil organic carbon (SOC), cation exchange capacity (CEC), pH, and particle-size were summarized in Leiva et al. [19].

**Table 1:** Selected soil properties and experimental setup for columns A, E, Bh, and multilayered column [A+E+Bh].

Property or Set-up	Parameters	Soil Horizon or Column			
		A	E	Bh	[A+E+Bh] ^k^
**Soil Physical & Chemical**	pH *	4.02	4.10	4.14	^NA^
	Sand (%)	93.8	97.2	96.4	^NA^
	Silt (%)	5.0	2.7	2.6	^NA^
	Clay (%)	1.2	0.1	1.0	^NA^
	CEC (cmol_c_ kg^-1^) ^a^	7.63	0.74	6.85	^NA^
	SOC (g g^-1^) ^b^	8.0×10^−3^	3.0×10^−3^	1.3×10^−2^	^NA^
**Column Packing**	*L* (cm)	15	15	15	[3, 8, 4]
	*m* (g) ^c^	976	1137	1085	[196, 608, 283]
	*ρ*_*B*_ (g cm^-3^) ^d^	1.47	1.72	1.64	[1.48, 1.72, 1.60]
	*ρ*_*S*_ (g cm^-3^) ^e^	2.56	2.65	2.61	[2.56, 2.65, 2.61]
	*θ* ^f^	0.43	0.35	0.37	[0.42, 0.35, 0.39]
**Input Pulse & Water Flow**	*C*_*o*_ (μg mL^-1^) ^g^	71	73	96	49
	*q* (cm min^-1^) ^h^	0.229	0.229	0.216	0.228
	*v* (cm min^-1^) ^i^	0.53	0.65	0.58	[0.54, 0.65, 0.58]
	*D* (cm^2^ min^-1^) ^j^	0.42	0.11	0.46	[0.09, 0.06, 0.12]
	Saturation volume (*τ*)	6.0	6.0	6.0	5.0
	Pulse length (*τ*)	4.31	2.88	4.37	2.23
	Total duration (*τ*)	16.2	12.4	15.0	11.5

* Soil pH in fertilizer mixture (soil:solution ratio of 1:4) after equilibration for 2 hr.

^a^ Cation exchange capacity.

^b^ Soil organic carbon.

^c^ Mass of oven-dry soil.

^d^ Oven-dry bulk density.

^e^ Particle density.

^f^ Volumetric water content at saturation.

^g^ IMD pulse solution concentration.

^h^ Water flow rate.

^i^ Pore water velocity.

^j^ Hydrodynamic dispersion coefficient.

^k^ Commas separate individual horizon values.

*τ* Pore volumes.

^NA^ Not applicable.

### Reagents and pulse solutions

IMD analytical crystalline standard (>99.5% purity) was obtained from ChemService Inc. (West Chester, Pennsylvania) and it was diluted in a stock solution of HPLC grade methanol obtain from Thermo Fisher Scientific (Pittsburg, Pennsylvania) of 1000 μg mL^-1^. Input-pulse solutions (*C*_*o*_) were prepared separately for each column experiment (A, E, Bh, and A+E+Bh), by diluting the stock with deionized water to concentrations of 71, 73, 96, and 49 μg mL^-1^, respectively (Table 1). The input concentrations were chosen based on IMD application rates normally used in Florida citrus groves (between 0.51 to 1.02 kg IMD ha^-1^ for Admire-Pro, Bayer CropScience). The higher concentration used in the Bh column was chosen to account for the higher soil organic carbon content in this soil, while the lower input concentration in the combined column was chosen based upon the E horizon’s dominance in these soils and its low sorption characteristics. The IMD input solution was prepared in a mixture containing nitrogen, phosphorous and potassium (NH_4_NO_3_, K_2_HPO_4_, and KCl; Thermo Fisher Scientific) that followed fertilization rates for Florida citrus [41]. Chloride (Cl^-^) solution was used as tracer for water flow and hydrodynamic dispersion. The rationale to apply input-pulses of IMD in a fertilizer mixture was to simulate field conditions (and soil solutions) as close as possible. The BTC data generated from the inorganic nutrients in the fertilizer mixture will be discussed in a separate paper.

### Sorption equilibria and sorption kinetics

Samples from the A and Bh horizons were used to determined IMD sorption kinetics (SK) and 24hr- sorption equilibria (SE) following procedures summarized in Leiva et al. [19]. The E horizon was not tested for SK nor SE due to its low organic matter content and negligible sorption for IMD [19]. Five grams of dry soil (*m*) were weighted in 50 mL polycarbonate centrifuge tubes (Nalgene, Thermo Scientific). The spiking solution volume (*v*) was 20 mL of three concentrations of IMD in fertilizer mixture: the initial input-pulse concentration (*C*_*o*_), and two dilutions of 0.5 *C*_*o*_ and 0.25 *C*_*o*_. Soil samples and solutions were equilibrated in an Eberbach horizontal shaker for 2, 4, 8, 12, 24, and 48 hours. Fertilizer mixture blanks (no soil added) were also equilibrated and analyzed to account for IMD sorption on container walls. After shaking, the tubes were centrifuged for 10 min at 6000 rpm. The supernatant or extract was filtered with Whatman 42 filter paper and refrigerated until analysis. Kinetics and equilibria data were analyzed following procedures detailed by Leiva et al. [19].

In this study, the sorption isotherms followed the Freundlich model. Also, the Freundlich sorption coefficient (*K*_*f*_) was linearized using the equation *K*_*D*_ = *K*_*f*_*[C*_*max*_*]*^*N-1*^, where *C*_*max*_ was the maximum concentration observed during the miscible displacement experiment [42].

### Miscible displacement experiments

The experiments were conducted at room temperature (22°C) in a clear Plexiglas column with an internal diameter of 7.5 cm and 15 cm of length (*L*). Each experimental column was dry-packed to the desired bulk density using a vibrator by filling 2.5 cm length sections at a time. Three separate miscible displacement experiments were conducted, one for each of the three IFS soil diagnostic horizons. Additionally, a column containing the three diagnostic horizons was prepared (Table 2, [A+E+Bh]) mimicking a representative IFS profile based on characterization data obtained from USDA-NRCS [37]. All column experiments were initially saturated with simulated Florida-rain without Cl^-^ [42], using a Gilson pump model 302 (Gilson Inc., Middleton, Wisconsin) with a pump-head with flow-rate capability between 1 and 10 mL min^-1^. Due to the coarse particle size distribution (sand contents >94%), it was possible to stablish similar flow rates for the different soil columns. The saturation step consisted on slowly applying (0.5 mL min^-1^) five to six pore volumes (Table 2) from the column bottom-inlet to allow air bubbles to exit at the top-outlet. At the same time, water flow was monitored with an analytical benchtop scale until steady-state Darcy flux *q* (cm min^-1^, Table 1) was achieved.

**Table 2:** HYDRUS-1D input parameters to simulate IMD transport in the multilayered Immokalee fine sand column under saturated water-flow.

Soil	Depth (cm)	Qr ^a^ (cm^3^ cm^-3^)	Qs ^b^ (cm^3^ cm^-3^)	Disp ^c^ (cm)	K_D_ (cm^3^ g^-1^)	Frac ^d^	Alpha ^e^ (min^-1^)
**A**	0–3	0.045	0.42	0.789	0.775	0	0.72
**E**	3–11	0.045	0.35	0.165	0.040	1	0
**Bh**	11–15	0.045	0.39	0.789	0.503	0	0.33

^a^ Residual water content, from van Genuchten model.

^b^ Saturated water content, from van Genuchten model.

^c^ Dispersivity = D/v.

^d^ Fraction of equilibrium or type-1 sites.

^e^ Mass transfer coefficient *α* in Eq 4.

The pulse-solution (IMD+fertilizer mixture) was transferred to amber bottles and applied to the columns using a switch-valve to change from saturation solution (Florida rain) to input solution. During column saturation, a fraction collector was calibrated to sample 20 mL of effluent in glass essay tubes, every 2 min. Once the input pulses were initiated, sample collection started, and subsequently, the tubes were capped with rubber stoppers to avoid evaporation from the sample. After the input-pulse was complete, the flow was switched back to Florida rain. The effluent samples were immediately transferred to 20 mL plastic scintillation vials and kept cool (4°C) in a refrigerator before analysis.

### Analytical methods

The HPLC calibration standards were prepared from a 1000 μg IMD mL^-1^ stock solution in HPLC grade methanol. The calibration standards were serially diluted with fertilizer mixture solution and showed good linearity (r^2^ = 0.999) in the range from 0.1 to 100 μg mL^-1^. Before HPLC analysis, effluent samples were re-filtered with Fisherbrand PTFE sterilized syringe filters (0.45 μm). The collected effluent samples were analyzed using an Agilent Infiniti 1260 HPLC-UV system (Agilent Technologies, Santa Clara, California), with a mobile phase of HPLC grade Acetonitrile-Water (40–60), a Supelcosil^™^ LC-18 column (150 x 4.6 mm, 5 μm particle size; Sigma-Aldrich Co.), an injection volume of 20 μL, 1 mL min^-1^ flow rate, and 270 nm of absorption wavelength. The analysis by HPLC-UV for the eluents had limits of detection and quantitation (LOD and LOQ) of 0.30 and 1.0 μg mL^-1^, respectively [19]. The retention time for IMD under these conditions was close to 2.7 minutes. Blanks of fertilizer mixture (non-spiked with IMD) were equilibrated and transferred to clean centrifuge tubes and spiked to 1.0 μg IMD mL^-3^ to estimate analytical recovery. There was no evidence of sorption on container walls, nor losses during filtration. The eluent samples for tracer (Cl^-^) analysis were processed using method 325.2 from the United States Environmental Protection Agency [43].

### Transport models and parameter optimization

The distribution coefficients obtained from batch kinetics and equilibria experiments, as well as the ones obtained from the column studies were compared to assess the effect of the experimental conditions on the transport parameters. The procedures to calculate the sorption coefficient (*K*_*D*_) from batch kinetics experiments were outlined previously [19], where the supporting electrolyte was 0.01 M CaCl_2_. For this study, the fertilizer mixture was used as the supporting electrolyte for the batch experiments. The IMD concentration range differ in one order of magnitude from our previous study [19] where a concentration range between was 2 to 8 μg mL^-1^, while these column studies used concentrations from 25 to 96 μg mL^-1^.

Our previous data on IMD sorption kinetics under batch conditions [19] were described by the one-site mass transfer kinetic model, which assumed sorption happened on kinetic or type-2 sites [21, 22, 44]. Therefore, in this study the OSNE-Model was used to describe IMD breakthrough curves from the A and Bh soil columns. The OSNE-Model is a special case of the two-site nonequilibrium (TSNE) model where the fraction of type-1 sites (instantaneous equilibrium) is zero, and type-2 sites are dominant. The dimensionless parameters of the OSNE-Model are essentially the same as the TSNE model, except for *β* and *ω* [21, 22, 44, 45]. The dimensionless form of the OSNE-Model and its parameters are shown in Eqs (1)–(4) without accounting for degradation, since we assumed that it was negligible during the short time of the column experiments:

$$\beta R\frac{\partial C^{*}}{\partial \tau }+\left(1-\beta \right)R\frac{\partial S^{*}}{\partial \tau }=\frac{1}{P}\frac{\partial^{2}C^{*}}{\partial X^{2}}-\frac{\partial C^{*}}{\partial X}$$ (1)

$$\left(1-\beta \right)R\frac{\partial S^{*}}{\partial \tau }=\omega \left(C^{*}-S^{*}\right)$$ (2)

$$\beta =\frac{1}{R}$$ (3)

$$\omega =\frac{\alpha K_{D}\rho_{b}L}{q}$$ (4)

where C* is the relative concentration in the liquid phase (*C/C*_*o*_), *R* is the retardation factor (1+ *K*_*D*_*ρ*_*b*_*/θ*), *K*_*D*_ is the average or linear soil partition coefficient (mL g^-1^), *ρ*_*b*_ is the bulk density (g cm^-3^), *θ* is the volumetric water content (cm^3^ cm^-3^), *v* is the pore water velocity (cm min^-1^), *D* is the hydrodynamic dispersion coefficient (cm^2^ min^-1^). The parameter *P* is the Peclet number (*vL/D*), *τ* represents dimensionless time in pore volumes (*vt/L*), and *X* is the relative transport distance in one-dimension (*x/L*). S* is the dimensionless sorbed concentration in type-2 sites [*S*/(*K*_*D*_ *C*_*o*_)], and *S* is the sorbed concentration (μg g^-1^). The parameter *β* is the fraction of instantaneous retardation to the total retardation [44], and *ω* is a Damköhler number [46] expressing the ratio of the reaction rate to the transport rate, based on the mass transfer coefficient *α* (min^-1^) and the Darcy flux *q* (cm min^-1^). However, when *α = 0 or β* = 1, the OSNE-Model in Eqs (1)–(4) reduces to the one-dimensional convective-dispersive model or CD-Model [21, 22]. The latter was used to describe the BTC of IMD in the E horizon, as well as the tracer BTCs for all four displacement experiments. The dimensionless form of the CD-Model and corresponding parameters describing solute transport in homogeneous porous media were described in detail by Skaggs et al. [47].

The BTCs for the tracer were simulated with the CD-Model by optimizing the *P* value as a measure of hydrodynamic dispersion, by keeping *R = 1* and *τ* constant, since the tracer was not adsorbed and the pulse was measured during the experiments. The IMD breakthrough curve from the E soil column was used to optimize *R* using the CD-Model, keeping *P* and *τ* values constant (from the tracer BTC). *R* and *ω* for the A and Bh soil columns BTCs were optimized using the OSNE-Model, again by keeping the values of *P* and *τ* used for the tracer. The OSNE-Model parameters were also fitted to the BTC of a simulated IFS profile [A+E+Bh] and were considered as “effective” parameters that could be used to simulate IMD transport in IFS soil profile. In addition, the optimized parameters for each horizon [A, E, and Bh] were used to describe IMD transport through the column [A+E+Bh].

### Multilayered column and transport simulations

A pulse-input applied to the column mimicking IFS soil profile (Table 1, [A+E+Bh]) was used to test the optimized parameters from each individual column (A, E, and Bh) to simulate IMD transport. The simulation was generated with HYDRUS-1D [33] and a summary of input parameters is presented in Table 2. Imidacloprid transport in the E horizon (which was described by the CD-Model) was indicated in HYDRUS-1D with an equilibrium state set by the fraction of type-1 sites *(Frac = 1)* and no kinetic mass transfer (*Alpha* = 0), specified in the HYDRUS-1D “*solute transport and reaction parameters*”. The simulation used was a “*standard solute transport”* with a *constant flux* of 0.228 cm min^-1^ for the whole column (or layers). The upper and lower *water flow boundary conditions* were set as “*constant pressure head*”. The transport model was the *“One-site sorption model (Chemical Nonequilibrium)”* and boundary conditions were specified as “*Concentration Flux BC*” for the upper boundary, and “*Zero Concentration Gradient*” for the lower boundary, with an input-pulse mode. The simulated BTC was specified with an observation node at 15 cm (column outlet).

### Statistical analysis

SigmaPlot 13 (Systat Software Inc., San Jose, California) was used to generate linear regressions for sorption isotherms. Analysis of covariance (ANCOVA) and Tukey’s HSD tests were used compare sorption coefficients between horizons. The transport model parameters were determined by nonlinear regression procedures in the STANMOD package [48, 49] which employs the CFITIM code to optimize the parameters of the governing models of solute transport in porous media. The goodness-of-fit of the optimized CD- and OSNE- transport models were evaluated using absolute error differences (AE), root mean squared error (RMSE), and correlation (r^2^) between the observed and fitted values by the CD- and OSNE-models [50]. AE was defined as the sum of the *absolute* differences between observed and fitted relative concentration values (C*). RMSE was defined as the squared root of the sum of differences between observed and fitted values of C*, divided by the degrees of freedom (number of observations—number of fitted parameters). The Pearson correlation coefficient (r^2^) accounted for the total sum of squares explained by the optimized transport models [51].

## Results

### IMD sorption equilibria and sorption kinetics

Table 3 summarizes data on IMD 24-hr sorption equilibria (SE) which used the fertilizer mixture as background electrolyte in the samples from the A and Bh columns. No evidence of sorption on centrifuge tube walls nor glass test tubes (for effluent fraction collection) was observed. In the SE study, the higher concentrations used (12.0 to 96.0 μg mL^-1^) showed sorption coefficients described by the Freundlich model [52] in both the A and Bh horizon samples, with exponents N<1. The linearized Freundlich coefficients for the A and Bh horizons (1.00 ± 0.48 and 0.58 ± 0.15 cm^3^ g^-1^) showed no difference between estimates (Tukey test, p>0.32), a result that agreed with our previous findings on IMD 24 hr. sorption equilibria [19]. The *K*_*D*_ values obtained from the BTC experiments and used in the HYDRUS-1D simulations (Table 4) were smaller when compared to the ones obtained from SE and SK experiments (Table 3). This result was attributed to the higher water flux or convective nature of the BTC experiments (*q*, Table 1) and the little organic matter in these soils, which ultimately reduced IMD retardation and sorption during the BTC experiment.

**Table 3:** Imidacloprid sorption equilibria and kinetics parameters (and 95% confidence intervals) in Immokalee fine sand samples from A and Bh horizons. Capital letters indicate differences between horizons.

Experiment	Parameters	A horizon	Bh horizon
**Sorption Equilibria** **24-hr batch**	*K*_*f*_ (cm^3^ g^-1^) ^a^	1.82 (0.87) ^A^	1.40 (0.37) ^A^
	*N* ^b^	0.86 (0.12) ^A^	0.80 (0.06) ^A^
	*K*_*D*_ linearized	1.00 (0.48) ^A^	0.58 (0.15) ^A^
**Sorption Kinetics** **2, 4, 8, 12, 24, 48-hr**	*C*_*o*_ (μg cm^-3^)	71	96
	*α* (min^-1^)	0.01 (0.003) ^A^	0.01 (0.01) ^A^
	*K*_*D*_ (cm^3^ g^-1^) ^c^	1.06 (0.05) ^A^	0.62 (0.02) ^B^

^a^ Freundlich coefficient.

^b^ Freundlich exponent.

^c^ Partition coefficient optimized with the one-site kinetic mass transfer model [19].

**Table 4:** Goodness-of-fit for the CD- and OSNE- transport models describing the tracer (Cl^-^) and Imidacloprid (IMD) breakthrough in Immokalee fine sand single-layer columns (A, E, Bh) and multi-layered [A+E+Bh] column.

Soil Column	Samples or Data Pairs ^c^	CD-Model (Cl^-^)			OSNE-Model (IMD)		
		AE ^d^	RMSE ^e^	r^2^ ^f^	AE	RMSE	r^2^
A	34 (51)	1.36	0.06	0.98	0.64	0.02	0.99
E ^a^	32 (47)	0.67	0.05	0.99	0.88	0.03	0.99
Bh	33 (45)	0.78	0.04	0.99	0.91	0.02	0.99
[A+E+Bh] ^b^	49 (40)	1.87	0.06	0.98	1.12	0.04	0.99

^a^ Imidacloprid BTC was fitted with the CD-Model.

^b^ OSNE effective parameters (Fig 5).

^c^ Number of samples analyzed for tracer (IMD in brackets).

^d^ Sum of C/C_o_ absolute errors.

^e^ Root mean square error.

^f^ Pearson correlation coefficient.

Sorption kinetics (SK) parameters *α* and *K*_*D*_ followed the one-site kinetic mass transfer model [19]. The SK coefficients for A and Bh samples were essentially the same as the ones obtained from the SE experiments (Table 3). SK data did show a significant difference between the sorption coefficients of A and Bh soil samples (p = 0.021). The lower sorption in the Bh horizon agreed with our previous findings on IMD sorption kinetics in sandy soils [19].

### IMD nonequilibrium transport in single-layer columns

**Fig 2:**
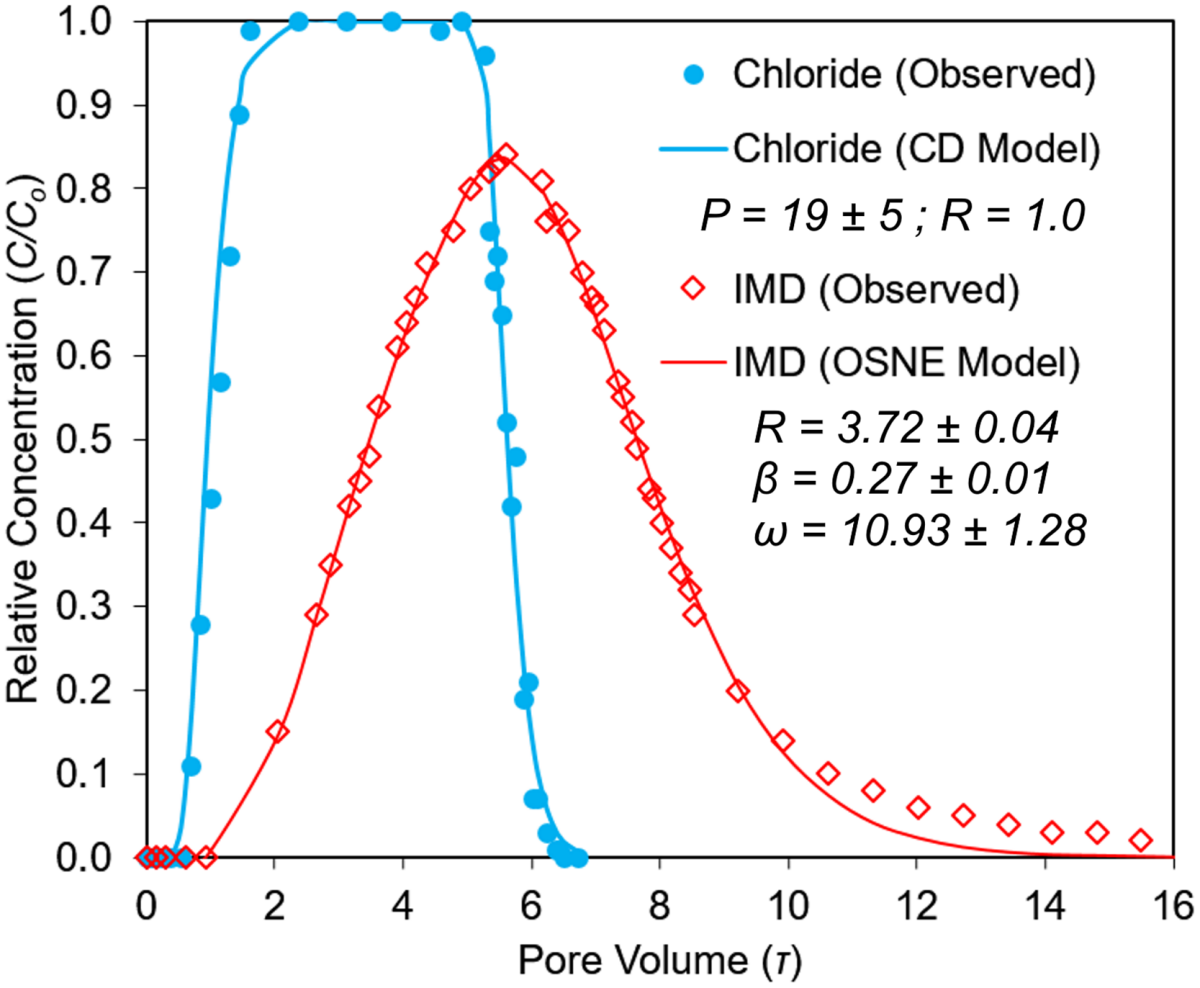
Imidacloprid (IMD) and tracer (Cl^-^) BTC from the A-horizon column and corresponding transport parameters. Solid lines indicate the optimized transport models (CD and OSNE).

**Fig 3:**
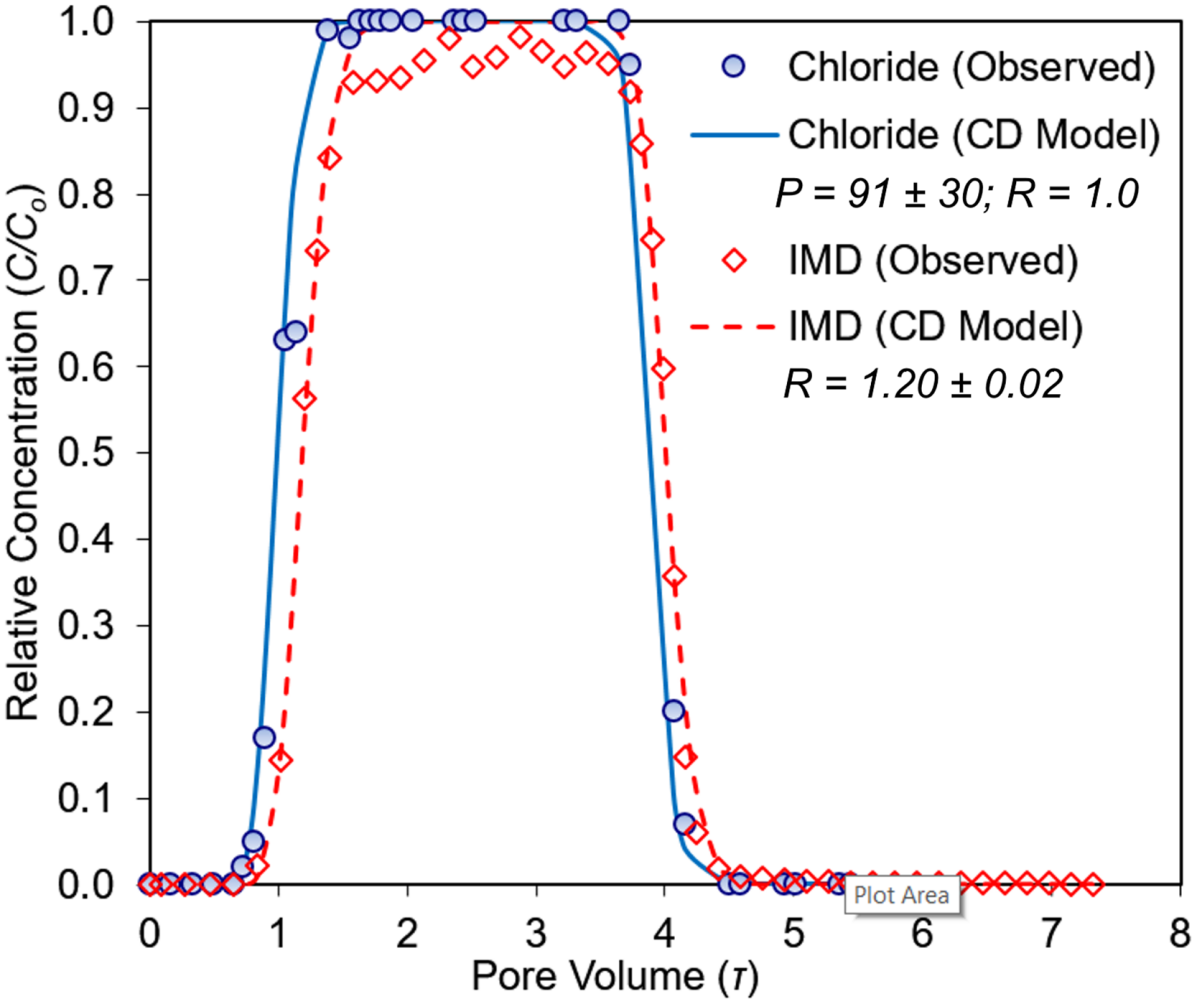
Imidacloprid (IMD) and tracer (Cl^-^) BTC from the E-horizon column and corresponding transport parameters. Solid lines indicate the optimized transport models (CD).

**Fig 4:**
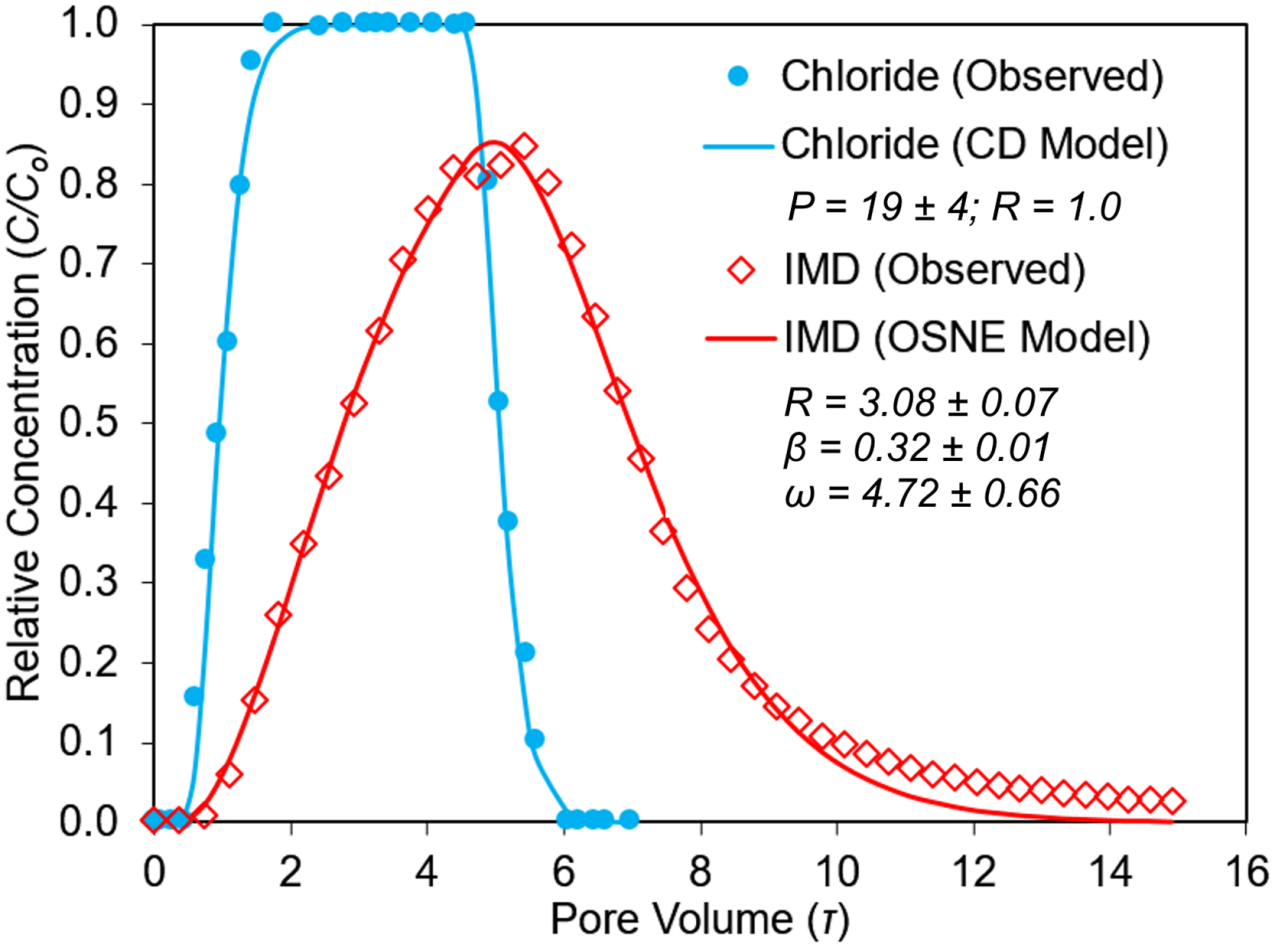
Imidacloprid (IMD) and tracer (Cl^-^) BTC from the Bh-horizon column and corresponding transport parameters. Solid lines indicate the optimized transport models (CD and OSNE).

**Fig 5:**
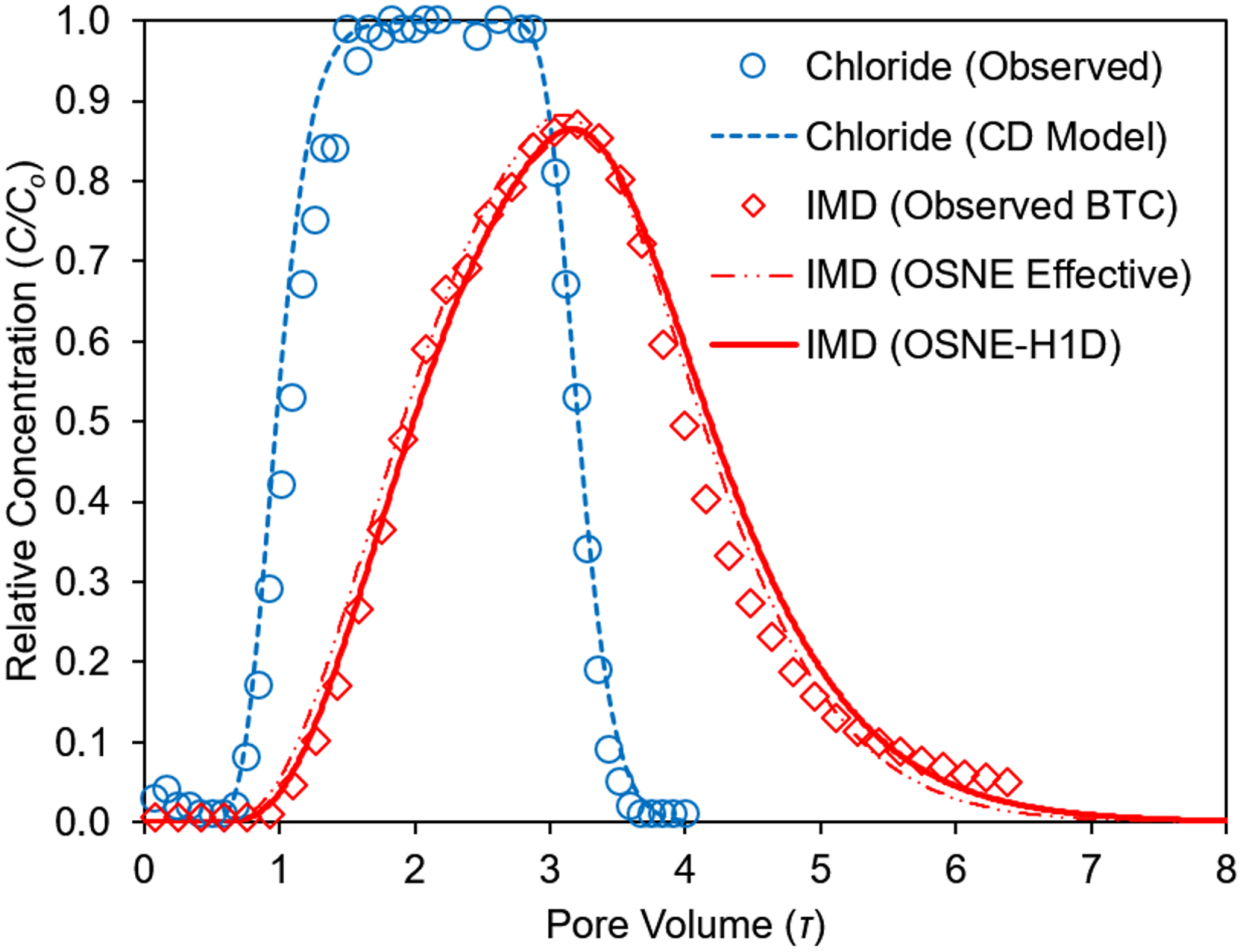
Imidacloprid (IMD) and tracer (Cl^-^) BTC from a multilayered IFS soil column [A+E+Bh]. HYDRUS-1D simulations used the effective parameters (OSNE Effective) from the multilayered [A+E+Bh] column, and the parameters from each soil horizon (OSNE-H1D).

The tracer and IMD BTCs were properly fitted by the CD-Model and OSNE-Model (Figs 2–4). In general, both transport models showed good correlations (r^2^ >0.99, p<0.02) and goodness-of-fit (RMSE <0.06), as summarized in Table 4. The average absolute differences between modeled and observed values were small: less than 1% for the tracer’s BTCs, and less than 2% for IMD’s (Table 4). The BTCs for the tracer (Cl^-^) in all experimental columns (single- and multilayered-) were characteristic of nonreactive-conservative tracers, with symmetrical shape and almost 100% mass recovery. The tracer BTCs had piston-displacement shapes and were properly described by the CD-Model (Figs 2–5). There were no evidence of physical nonequilibrium (regions of mobile-immobile water) in these soil columns [29]. Also, the tracer BTCs had relatively large *P* values (Table 5) that confirmed the homogeneity and convective nature of the porous media and the solute transport phenomena in these soils.

**Table 5:** CD-Model (*P*, *R*) and OSNE-Model (*R*, *β*, *ω*) optimized dimensionless parameters (± 95% confidence interval) for IMD and tracer (Cl^-^) in Immokalee fine sand. Letters (lowercase for A horizon, uppercase for Bh) indicate differences between the parameters obtained from the column and sorption experiments.

Experiment	Soil	*P* ^2^	R ^3^	*β*	*ω* ^5^
**Column** **Transport**	A	19 ± 5	3.72 ± 0.04 ^a^	0.27 ± 0.01 ^a^	10.93 ± 1.28 ^a^
	E	91 ± 30	1.20 ± 0.02	NA ^4^	NA
	Bh	19 ± 4	3.08 ± 0.07 ^A^	0.32 ± 0.01 ^A^	4.72 ± 0.66 ^A^
	[A+E+Bh] ^1^	46 ± 10	2.03 ± 0.04	0.49 ± 0.01	4.97 ± 0.75
**Sorption Equilibria & Kinetics**	A	NA	4.43 ± 1.64 ^b^	0.23 ± 0.13 ^b^	0.87 ± 0.04 ^b^
	Bh	NA	3.55 ± 0.67 ^A^	0.28 ± 0.07 ^A^	0.84 ± 0.03 ^B^

^1^ Effective transport parameters from the multilayered column (P, R, *β*, *ω)*.

^2^ Peclet number (P) from tracer BTC.

^3^ Retardation factor (R).

^4^ Not applicable (NA).

^5^ Dimensionless mass transfer coefficient (Eq 4).

Imidacloprid showed similar retardation and tailing in both the A and Bh columns and were adequately described by the OSNE-Model (Table 4). The optimized *ω* values were 10.93 ± 1.28 (95% confidence interval) for the A horizon (α = 0.14 min^-1^) and 4.72 ± 0.66 for the Bh horizon (α = 0.09 min^-1^). Therefore, the A column showed a larger Damköhler number, which implies a larger ratio of IMD reaction to transport rates between the solution and the soil, when compared to the Bh column. Moreover, IMD showed less retardation in the Bh horizon (*R* = 3.08 ± 0.07) than the A horizon (*R* = 3.72 ± 0.04), a result that agreed with our previous experiments on IMD 24 hr.-batch sorption, where the insecticide showed lower sorption in the Bh soil samples, in general [19].

The tracer BTC in E horizon column was described by the CD-Model, with a large Peclet number (*P* = 91, Table 5), indicating that the porous media was dominated by convective flow. Imidacloprid BTC was also described by the CD-Model, which implies that IMD had negligible sorption in the E column (Fig 3). Imidacloprid was weakly sorbed in this horizon, showing an *R* value of 1.20 ± 0.02. The slight difference between the tracer and IMD breakthrough in the E horizon (Fig 3) column was due to the little soil organic C content (0.3%). IMD transport parameters related to sorption (*R*, *β*) obtained from column BTCs and batch SE data were not statistically different (Tukey test, p>0.42, Table 5) for both the A and Bh soils. The exception was the mass transfer coefficient (*α*), where the column-derived estimates were larger than the *α* from the SK data (Table 3). This was due to the effect of the large Darcy flux used in these column BTCs and the corresponding effect on the mass transfer coefficient *α* (Eq 4).

### IMD transport in multilayered-column and BTC simulations

The observed BTC for IMD from the multi-layered column (Fig 5) also showed retardation and tailing and was properly described by the OSNE-Model (r^2^ = 0.99, p = 0.014). The IMD observed and fitted curve generated with the effective OSNE parameters had good agreement and small absolute errors estimates (<4% for tracer and IMD BTCs). The effective *P* for the multilayered column (Table 4) was higher than the individual A and Bh columns, due to the dominance (thickness) of the E layer. The effective *R* for IMD (*R* = 2.03) was lower than the values in the single-layer columns A and Bh, again attributed to the dominance of the E layer. The HYDRUS-1D transport simulations in this column had good agreement with the observed data, as well (Fig 5). The transport simulation using the OSNE effective parameters showed essentially the same BTC when compared to the simulation using parameters optimized from the single layer columns (Fig 5, OSNE-HD1 line). Based on these data, IMD transport in these soils was dominated by the effect of the E horizon, that decreased the overall retardation and sorption of the pesticide. The observed trend of IMD transport and nonequilibrium sorption agreed with published data for other neonicotinoids that showed low sorption in soils and moderate to high leaching potential [3–5, 16, 17].

## Discussion

In general, sandy soils are prone to contaminant leaching due to their macroporosity and low organic matter content. Since neonicotinoids are highly persistent in soil and very soluble in water [3, 53], their potential for leaching in sandy soils is high when compared to other soil textures. This has been the general result of laboratory and field studies of TMX [53–55] and IMD [16, 18, 53]. Based on the BTC data described in this study, IMD would have a moderate-to-high leaching potential in Immokalee fine sand, under worst-case scenario conditions. IMD sorption in the E horizon was essentially negligible (Table 4; Fig 3). However, IMD was moderately sorbed in the A and Bh soil horizons. IMD soil-drench applications to control ACP in soils of similar characteristics could be lost to leaching once IMD reaches the E horizon.

In this regard, a careful monitoring of soil moisture content in the citrus root-zone (mainly concentrated in the A horizon) by irrigation practices is crucial. Currently, low-volume irrigation systems used in citrus, such as micro-sprinkling and drip-systems are excellent choices for keeping the soil moisture low but sufficient to satisfy crop needs [56, 57]. Also, these irrigation systems maintain the soil in mostly unsaturated conditions, that in theory should increase retardation factors and residence times, even for weakly-sorbed pesticides such as IMD. Consequently, increasing the residence time of IMD in the root-zone should enhance plant uptake and reduce the potential for IMD leaching to groundwater in Flatwoods areas of Florida under citrus production.

Sorption nonlinearity is common in most interactions between hydrophobic organic contaminants in soil-solution and the soil organic matter [52, 58]. The complex nature of soil surfaces (biofilms, organic matter, clays, etc.) generate chemical and physical nonequilibrium in the sorption and transport phenomena [29, 52]. In this study, IMD sorption equilibria was nonlinear and followed the Freundlich isotherm model (with exponents N<1) which indicated lower sorption rates at higher concentrations in solution [52]. IMD sorption will be highest when present at lower concentrations in the soil solution after drenching in these soils. Therefore, a general recommendation is to drench IMD in several splits of the recommended label rate. This practice should increase IMD sorption and retention times, and will enhance root uptake and ACP control programs.

The data trend of lower IMD sorption and/or retardation in the spodic layer (Bh column) was previously noted by our sorption and kinetics results [19, 20], and could be related to one or more of the following: a) The podzolization process of IFS which has removed Fe or Al from the A and E horizons, translocating them as organic chelates to the Bh layer. The organo-chelate reactions could have generated an organic matter fraction with different affinity (or sorption) for IMD. b) The higher silt and clay contents of the Immokalee fine sand A horizon may have increased IMD sorption under batch and transport conditions, a result that agrees with previous work by Fernandez-Bayo et al. [59] who found the same trend for IMD in soils from Southern Europe with low organic matter contents.

In conclusion, Imidacloprid transport in Immokalee fine sand (IFS) showed weak-to-moderate sorption and significant evidence of nonequilibrium transport in soil columns packed with A, Bh, and [A+E+Bh] horizons from IFS. The findings agreed with previous research on neonicotinoid transport in soils, where leaching and pollution of groundwater is a high concern [3, 17, 19, 20, 60]. Since IMD is normally soil-drenched to sandy soils of Florida during control programs for ACP, the pesticide could be lost to leaching once it passes the A horizon (where most of the citrus roots concentrate) and enters the E horizon where retardation is negligible. Field leaching studies are necessary to evaluate the transport parameters optimized from our column experiments, and to test drench and irrigation rates, and to analyze the effect of transient soil moisture conditions on IMD leaching. Current irrigation systems used in citrus groves of Florida, such as micro-sprinkling and drip irrigation have been designed to maintain moisture contents that satisfy citrus water requirements and growth [56]. Theoretically, when these soils are kept at or below field capacity (volumetric water contents around 0.09–0.10), they develop unsaturated conditions that would considerable increase IMD retention. Longer sorption in the citrus root-zone should enhance IMD plant uptake after soil-drench applications. Therefore, even in these sandy soils, keeping the root zone unsaturated will reduce the chance of leaching or pollution of groundwater.
